# Cr(VI) Removal by Recombinant *Escherichia coli* Harboring the Main Functional Genes of *Sporosarcina saromensis* M52

**DOI:** 10.3389/fmicb.2022.820657

**Published:** 2022-03-03

**Authors:** Qiuying An, Min Zhang, Dongbei Guo, Guangshun Wang, Hao Xu, Chun Fan, Jiayao Li, Wei Zhang, Yi Li, Xiaoxuan Chen, Wanting You, Ran Zhao

**Affiliations:** ^1^State Key Laboratory of Molecular Vaccinology and Molecular Diagnostics, School of Public Health, Xiamen University, Xiamen, China; ^2^Huzhou Center for Disease Prevention and Control, Huzhou, China; ^3^National Cancer Center/National Clinical Research Center for Cancer/Cancer Hospital & Shenzhen Hospital, Chinese Academy of Medical Sciences and Peking Union Medical College, Shenzhen, China

**Keywords:** *Sporosarcina saromensis* M52, recombinant bacteria, hexavalent chromium, Cr(VI) reduction, bioremediation

## Abstract

Hexavalent chromium [Cr(VI)], a recognized heavy metal pollutant, has attracted much attention because of its negative impact on the ecological environment and human health. A chromium-resistant strain, *Sporosarcina saromensis* M52, was discovered, and the functional genes orf2987, orf3015, orf0415, and orf3237 were identified in the strain by genomics. With the advancement of DNA recombination and gene-splicing technology, genetic engineering technology was used to produce recombinant strains 2987, 3015, 0415, and 3237. The study revealed Cr(VI) tolerance in the order of M52 ≈ 2987 > 3015 ≈ 0415 > 3237 and reduction abilities in the order of M52 ≈ 2987 > 3015 > 0415 ≈ 3237. SEM-EDS, XRD, FT-IR and XPS were utilized to examine the surface structure of the recombinant strains and analyze the surface components and main functional groups. A comprehensive review of the recombinant strains’ capacity to tolerate and reduce Cr(VI) revealed that orf2987 and orf0415 were the main functional genes in *Sporosarcina saromensis* M52, which may play a key role in removing Cr(VI) and protecting the strain, respectively. The optimum pH for recombinant strains 2987 and 0415 was 7.5–8.5, and the optimum temperature was 37°C. Cu^2+^ had the greatest promotional effect when Cr(VI) was removed by them, while SDS had an inhibitory effect. This research provided the foundation for further study into the mechanism of Cr(VI) reduction in *Sporosarcina saromensis* M52, as well as a theoretical basis for the development of effective engineered strains to repair Cr(VI) contamination.

## Introduction

With the rapid development of industry, chromium (Cr) has been widely used in electroplating, wood preservation, material dying, leather tanning and other industries, and associated industrial effluents pose a serious threat to ecology and human health (Stern, 2010; Malaviya and Singh, 2016; Diaconu et al., 2020). The oxidation state of Cr varies from −2 to +6, in which trivalent chromium [Cr(III)] and hexavalent chromium [Cr(VI)] are the main forms in water (Thatoi et al., 2014). Cr(III) is a necessary trace element for the human body (Karthik et al., 2017; Ma et al., 2019), whereas Cr(VI) has been proven to have carcinogenicity, teratogenicity, and mutagenicity (Xia et al., 2019). Since the toxicity of Cr(VI) is 100 times higher than that of Cr(III) (Xia et al., 2019; Gu et al., 2020), converting Cr(VI) to Cr(III) becomes the main strategy to address Cr(VI) pollution.

The main ways to reduce Cr(VI) to Cr(III) are physical, chemical, and biological remediation methods (Nayak et al., 2020). Many physical and chemical methods, such as membrane separation, chemical precipitation, ion exchange, and electrochemical methods, are currently used, but they often have the disadvantages of being expensive, taking a long time, requiring a large amount of reagents, and eventually producing a large number of secondary pollutants that are difficult to deal with (Agrawal et al., 2006; Barrera-Diaz et al., 2012; Bvrith and Reddy, 2013; Jobby et al., 2018). However, when compared to these traditional physical and chemical remediation methods, bioremediation has the unique advantages of being simple to operate, low in cost, requiring fewer reagents, and producing fewer secondary pollutants, giving it the potential to become an environmentally friendly chromium remediation technology (Harish et al., 2012; Bharagava and Mishra, 2018). Many strains, such as *Bacillus*, *Pseudochrobactrum*, *Brevibacterium*, *Stenotrophomonas*, *Vigribacillus*, *Ochrobactrum*, and *Cellulosimicrobium* strains (Karthik et al., 2017; Banerjee et al., 2019), have been discovered to be able to tolerate and decrease Cr(VI) in recent decades. *Sporosarcina saromensis* M52 (M52), collected in Xiamen, also has the ability to effectively decrease Cr(VI), although the mechanism was unclear (Zhao et al., 2016). In previous studies, the functional genes of M52 were identified by genomic analysis (Li et al., 2021), which identified orf2987, orf3015, orf0415, and orf3237 as the major functional genes in M52, mainly responsible for reducing Cr(VI), but whether they really have the function of reducing Cr(VI) has not been confirmed.

With the advancement of DNA recombination and gene-splicing technology, genetic engineering technology can be used to construct recombinant strains that can verify the functions of orf2987, orf3015, orf0415, and orf3237. In the process of constructing, *Escherichia coli* BL21 (*E. coli*) is a suitable carrier for the following reasons: (1) *E. coli* has a very defined genetic background and has been authorized by the FDA as a safe genetically engineered recipient organism. (2) The culture technique for *E. coli* is simple, with rapid growth, a short culture period, good anti-pollution ability, and a low cost. (3) It has a mature and complete expression system capable of efficiently expressing the target protein (Ke et al., 2018). (4) It is the most widely used strain in research and industry and has been extensively studied (Jaen et al., 2019). (5) The reduction capacity of Cr(VI) is very weak (Guo et al., 2012).

In this research, the recombinant strains were created to verify and compare the reduction characteristics and tolerance of orf2987, orf3015, orf0415, and orf3273 in M52 to Cr(VI), as well as to screen out the key functional genes that play reducing and protective roles in M52. *Via* a laboratory simulation of the Cr(VI)-contaminated environment, the optimum conditions of recombinant strains containing key functional genes were determined for improved Cr(VI) reduction, and their functional groups in the reduction process were also detected. This research not only confirmed that orf2987, orf3015, orf0415, and orf3237 in M52 had reduction characteristics and tolerance to Cr(VI) but also provided the foundation for future research into the mechanism of reducing Cr(VI) in *Sporosarcina saromensis* M52, as well as a theoretical basis for the creation of engineering strains with high efficiency to remediation Cr(VI) pollution.

## Materials and Methods

### orf 2987, orf3015, orf0415, and orf3237 Genes

M52 was inoculated in LB medium at 37°C and cultivated at 200 rpm for 15 h after being revived. The TIANamp Bacteria DNA Kit was used to extract the genome. After extracting the M52 genome, BLAST p was aligned and annotated by the Nr, Swiss-Prot, Pfam, String, COG/KOG, KEGG, and GENE Ontology databases, and the orf2987, orf3015, orf0415, and orf3237 gene sequences were obtained. Special PCR primers (Supplementary Table S1) for cloning the orf2987, orf3015, orf0415, and orf3237 genes were designed by Primer Premier 5.0 and synthesized by Sangon Biotech (Shanghai, China). In the PCR experiment, we used *2× Pfu* fidelity mixing enzyme (Tiangen Biotech Co., Ltd., Beijing, China) to ensure correct cloning of the gene series. All PCR-amplified products were separated and identified by 1.5% agarose gel electrophoresis and recovered by the TIANgel Universal DNA Purification Kit (Tiangen Biotech Co., Ltd., Beijing, China).

### Construction of Recombinant Strains

In this experiment, pET-30a(+), which containing kanamycin resistance gene, was used as the expression vector for recombinant strains. The plasmid was extracted by the TIANprep Mini Plasmid Kit (Tiangen Biotech Co., Ltd., Beijing, China). The target genes orf2987, orf3015, orf0415, and orf3237 were subjected to double enzyme cutting by restriction enzymes (listed in Supplementary Table S1). After purification and connection, the orf2987, orf3015, orf0415, and orf3237 genes were combined with pET-30a(+) to produce recombinant vectors pET-2987, pET-3015, pET-0415 and pET-3237. Recombinant vectors were converted into *E. coli* BL21 (DE3) to create recombinant strains 2987, 3015, 0415, and 3237. Positive clone plasmids screened with a solid selective LB plate containing 50 μg/ml kanamycin were extracted and then confirmed by enzyme digestion and sequencing.

### Expression and Localization of Recombinant Proteins

Recombinant strains 2987, 3015, 0415, and 3237, were grown in LB medium with kanamycin (50 μg/ml) at 37°C. The strains were centrifuged at 4,000 rpm for 10 min, the supernatant was discarded, and the bacteria were suspended with 500 μl of bacterial lysate. After being washed and disrupted with 50 mM Tris–HCl, the strains were disrupted by ultrasonication for a total of 6 min (300 W, ultrasound for 0.5 s, period for 5 s) in an ice bath and centrifuged at 12,000 rpm for 20 min at 4°C. Supernatant and precipitate were used as samples where precipitation was dissolved in a 500 μl inclusion body solution (8 mM urea, 50 mM Tris–HCl, 300 mM NaCl, pH 8.0). Expressed recombinant proteins were identified by 10% sodium dodecyl sulfate-polyacrylamide gel electrophoresis (SDS-PAGE).

### Cr-Resistance and Cr-Reduction Characteristics of Recombinant Strains

#### Growth Curve of Strains

M52 and recombinant strains were cultured overnight for 14–16 h at 37°C and 200 rpm. The culture was then stopped and the seed liquid was prepared. The cultured 4% v/v seed liquid was inoculated into LB medium with and without Cr(VI). At different times, the A600 of the bacterial liquid was calculated by a full-wavelength enzyme labeling instrument, and the growth curve was drawn under Cr(VI)-free and Cr(VI)-containing conditions.

#### Resistance to Cr(VI)

M52 and recombinant strains were shaken and cultured at 37°C overnight. The strains were obtained by centrifugation and calibrated to the same concentration as A600. After washing with PBS three times, the strain was resuspended in sterilized LB medium containing various concentrations of Cr(VI) (0, 50, 100, 200, 400, and 800 mg/L) at pH 8.0 and cultured at 37°C and 200 rpm for 72 h. The A600 of the bacterial solution was tested every 12 h, and a growth curve was drawn to determine the Cr(VI) tolerance of the strains.

#### Reduction of Cr(VI)

After cultivating strains in sterilized LB medium containing various concentrations of Cr(VI) (0, 50, 100, 200, 400, and 800 mg/L), the colonies were centrifuged at 12,000 rpm at 4°C for 20 min twice. Supernatants were purified using 0.22 μm filters (Millipore, United States) to eliminate the remaining bacterial cells. The Cr(VI) content of the supernatant of the culture medium at different times was calculated by the diphenylcarbazide spectrophotometry method. At the beginning of the experiment, the A540 of the solution containing Cr(VI) was recorded as A_0_, the A540 of the solution to be evaluated was labeled as A_1_, and the A540 solution without Cr(VI) was recorded as C. The reduction percentage of the strain to Cr(VI) at the time to be tested was calculated by the following formula:


$$X=\frac{A_{0}-A_{1}}{A_{0}-C}\times 100\%$$

### Characterization of Cr(VI) Reduction

#### SEM-EDS Analysis

The surface morphology of the strains before and after the reduction of Cr(VI) was characterized by the use of a ZEISS Sigma Scanning Electron Microscope (SEM) fitted with an Energy Dispersive spectroscopy (EDS). The strains were cultivated in sterilized LB medium (pH 8.0) with 100 mg/L Cr(VI) at 37°C for 48 h. The strains were washed three times with 0.1 M PBS, and the suspension was scattered over a glass slide and dried. Samples of immobilized strain were prepared according to the method of Garg et al. (2013). After spraying the gold, position the sample into the SEM-EDS unit, examine the different sections of the sample under an accelerating voltage of 15 kV, and then take micrographs under appropriate magnification.

#### XRD, FT-IR, and XPS Analysis

Fourier Transform Infrared Spectroscopy (FT-IR) and X-ray photoelectron spectroscopy (XPS) were used to record the process of chemical modification of strains before and after reduction of Cr(VI) to study the functional group responsible for reducing the surface area of strains. X-ray diffraction (XRD) analysis was performed on the control and Cr(VI)-treated strains to identify the crystallized Cr(III) species during the Cr(VI) reduction process. The strains were cultured in sterilized LB medium in the presence and absence of 100 mg/L Cr(VI). After 48 h of incubation, the cultivated strains were washed with PBS, extracted by centrifugation, and then lyophilized in a vacuum freezer dryer (Ma et al., 2019). FT-IR, XPS and XRD were all performed at the SLIntelligent Analysis Testing Center (Nanjing, China).

### Factors Affecting Cr(VI) Reduction

#### pH and Temperature

The effects of various pH values (7.0, 7.5, 8.0, 8.5, and 9.0) and incubation temperatures (25, 30, 35, 40, and 45°C) on Cr(VI) reduction in the strains were tested with 100 mg/L Cr(VI). Using a factorial design, each temperature level and pH level were completely randomly combined, and there were 25 groups in total, with three parallel samples in each group. All centrifuge tubes were placed in a constant temperature shaker at 200 rpm for 48 h. The culture was removed from each treatment at 12, 24, and 48 h and centrifuged twice at 4°C and 12,000 rpm for 20 min. The supernatant was then screened with a 0.22 μm filter (Millipore, United States) to eliminate the remaining bacterial cells. The Cr(VI) content of the culture supernatant was determined at different times to measure the Cr(VI) reduction percentage.

#### Metal Ions and Small Molecules

Cultured 4% v/v seed liquid was inoculated into LB medium containing 100 mg/L Cr(VI) with 0.2 mM Mn^2+^, Fe^2+^, and Cu^2+^, which were all made with sulphates, and 0.1 mM SDS. The 100 mg/L Cr(VI) bacterial solution without metal ions and small molecular compounds was used as a control. All centrifuge tubes were cultured at pH 8.0, 37°C and 200 rpm for 48 h, and the cultures were regularly separated from each procedure every 12 h and centrifuged at 12,000 rpm for 20 min. The supernatant was then filtered with a 0.22-μm filter (Millipore, United States) to remove the remaining bacterial cells. The Cr(VI) content in the culture supernatant was determined at different times to ascertain the Cr(VI) reduction percentage.

### Statistical Analysis

Statistical analysis of all experimental results was performed using SPSS 23.0 test, where *p* < 0.05 indicates that the discrepancy is statistically important. Because the experimental data for resistance and reduction results for Cr(VI) recombinant strains were tested from multiple measurements of the A600 and Cr(VI) reduction rates at different time points while the strains were exposed to different concentrations of Cr(VI), repeated measurement analysis of variance was chosen to investigate the difference in the overall change trend of the data. Factorial design may construct the cross-grouping of many factors and investigate the variations between each factor level and the interaction between factors, which satisfy our requirements. Therefore, the impact of pH and temperature on Cr(VI) reduction by the recombinant strains was tested through a factor design analysis of variance. The effects of metal ions and small molecules were tested *via* single-factor analysis of variance.

## Results and Discussion

### Construction of Recombinant Strains

**Figure 1:**
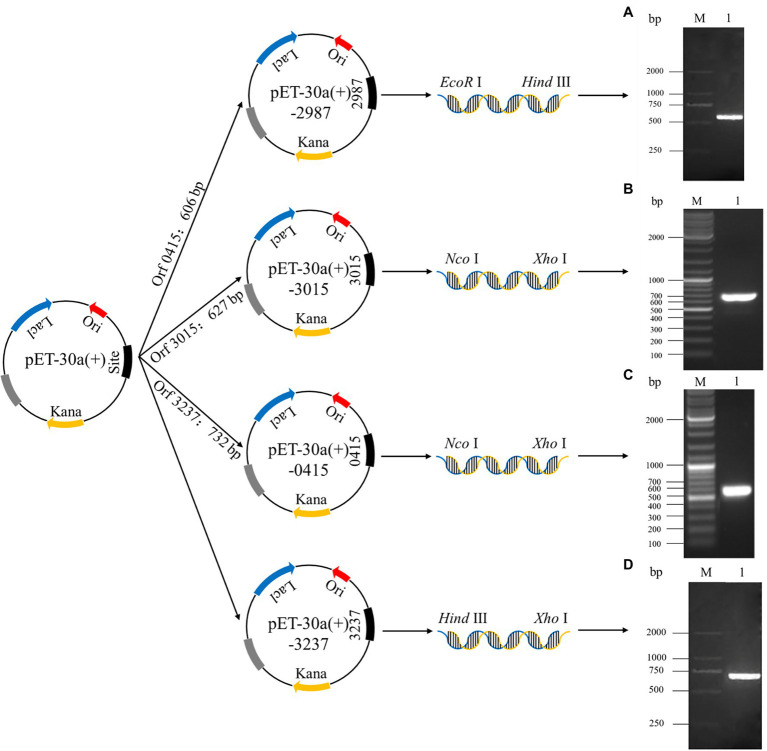
The establishment process of recombinant plasmids pET-30a (+)-2987, pET-30a (+)-3015, pET-30a (+)-0415 and pET-30a (+)-3237 and the analysis of the PCR products of target genes by 1.5% agarose gel electrophoresis. M: Marker. 1: Target genes 2987 **(A)**, 3015 **(B)**, 0415 **(C)** and 3237 **(D)**.

The construction process for recombinant plasmids is shown in Figure 1, and the PCR products of the recombinant strains were detected by 1.5% agarose gel electrophoresis. Agarose gel electrophoresis showed that target genes were successfully amplified from recombinant strains 2987, 3015, 0415, and 3237, with lengths of approximately 500, 650, 600, and 700 bp, respectively. The DNA sequencing results of recombinant strains 2987, 3015, 0415 and 3237 revealed that the sequences were the same as the target gene. Agarose gel electrophoresis results were strongly consistent with DNA sequencing results, indicating that the target gene was accurately linked to and transformed with the vector plasmid, and the recombinant strains had been constructed successfully.

### Expression and Localization of Recombinant Proteins

**Figure 2:**
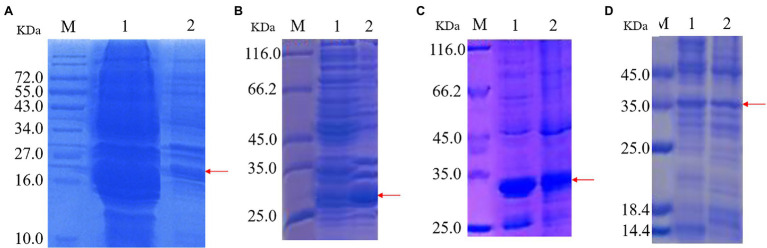
SDS-PAGE of protein expression induced by recombinant strains 2987 **(A)**, 3015 **(B)**, 0415 **(C)** and 3237 **(D)**. M: Marker. 1: Supernatant 2: Precipitate.

Recombinant protein expression is shown in Figure 2. Protein bands of approximately 22, 28, 30, and 35 kDa were apparent in recombinant strains 2987, 3015, 0415, and 3237, respectively. The results demonstrated successful induction and expression of recombinant proteins. However, the molecular weights of the proteins derived by SDS-PAGE were approximately 2 kDa larger than their theoretical values (2987: 20 kDa, 3015: 28 kDa, 0415: 28 kDa, and 3237: 34 kDa). The reason for this was that the His-tag molecular weight in the pET-30a (+) plasmid vector.

The target protein bands of all recombinant strains were detected in supernatant and precipitation, although in varied proportions. Target protein 2987 was primarily detected in supernatant, whereas target proteins 3015, 0415, and 3237 were found in a combination of supernatant and precipitation, but the protein concentration of 3015 and 3237 was much greater than that of 3237. Li et al. (Mengke et al., 2019) discovered that the primary components of supernatant were intracellular soluble chemicals, whereas the primary components of precipitation were cell membrane and insoluble proteins. Kaur et al. (2018) also discovered that high-level recombinant protein expression usually resulted in the accumulation of insoluble aggregated folded intermediates in the cytoplasm as inclusion bodies. To summarize, all recombinant proteins were soluble proteins, but some of them might exist as inclusion bodies because of overexpression. These results were consistent with previous genomic (Li et al., 2021) and proteome (data being submitted) analyses.

### Resistance to Cr(VI)

**Figure 3:**
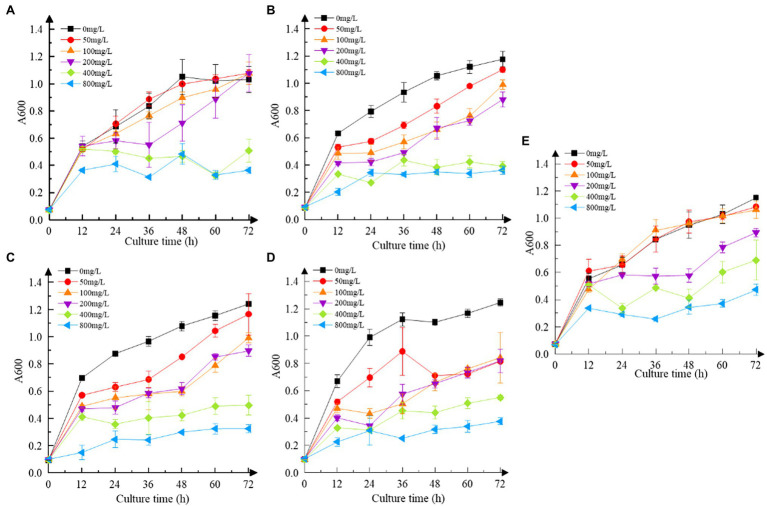
Cr(VI) resistance of strains. **(A)** Recombinant strain 2987; **(B)** recombinant strain 3015; **(C)** recombinant strain 0415; **(D)** recombinant strain 3237; and **(E)** M52 (control). Date represent means ± SD. All experiments were repeated three times.

As shown in Figure 3, the resistance of all strains to Cr(VI) followed the same pattern. As the concentration of Cr(VI) increased, the growth of the strains was impaired to some extent when compared to the Cr(VI)-free group, obtaining the same results as Igiri et al. (2018). At 400 mg/L and 800 mg/L, the growth of all strains was sluggish in comparison to those without Cr(VI) (*p* < 0.05). Clear signs of sluggish growth were found in recombinant strain 3237 at 50 mg/L Cr(VI), whereas in recombinant strains 3015 and 0415 at 100 mg/L Cr(VI), and in recombinant strain 2987 at 200 mg/L Cr(VI) same as M52 (*p* < 0.05). Furthermore, the growth of all strains at various concentrations of Cr(VI) is shown in Supplementary Figure S1. The results revealed that 800 mg/L Cr(VI) strongly inhibited the growth of all strains, indicating that the minimum inhibitory concentration of M52 and the recombinant strains was the same, which was 800 mg/L Cr(VI). However, at 100 mg/L Cr(VI), recombinant strains did not grow as well as M52 except 2987 (Supplementary Figure S1b; *p* < 0.05). These results indicated that the tolerance of the strains to Cr(VI) was M52 ≈ 2987 > 3015 ≈ 0415 > 3237, which suggesting that these four functional genes may play a protective role in M52 surviving, allowing the strain to tolerate Cr(VI), with orf2987 having the most protective effect. However, this protective effect might not be attributable to the fact that orf2987 was a chrome-resistant gene, but rather to its great capacity as a chrome-reduction gene, therefore, the specific gene function must be further confirmed by chromium reduction determination. Considering the growth of strains, 100 mg/L Cr(VI) was chosen as the intervention condition for the subsequent experiments.

### Reduction of Cr(VI)

**Figure 4:**
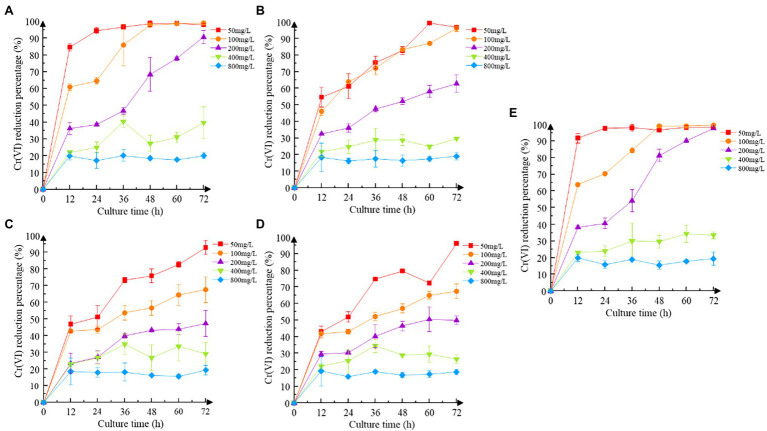
Cr(VI) reduction of strains. **(A)** Recombinant strain 2987; **(B)** recombinant strain 3015; **(C)** recombinant strain 0415; **(D)** recombinant strain 3237; and **(E)** M52 (control). Date represent means ± SEM. All experiments were repeated three times.

Increasing the concentration of Cr(VI) inhibited the reduction efficiency of recombinant strains to some extent (Figure 4). Recombinant strain 2987, which was identical to M52 in reduction efficiency, can completely reduce Cr(VI) at concentrations of 50 mg/L and 100 mg/L Cr(VI), but the reduction rate at 200 mg/L Cr(VI) was much lower (*p* < 0.05). While the reduction efficiency of recombinant strain 3015 was higher at low concentrations of Cr(VI) (50 and 100 mg/L), it was still not as good as 2987 (*p* < 0.05), and the other two recombinant strains began to show signs of inhibition at 100 mg/L Cr(VI) (*p* < 0.05). Except for 2987, the reduction percentage of M52 was higher than that of the recombinant strains in 50, 100 and 200 mg/L Cr(VI) (*p* < 0.05), as shown in Supplementary Figure S2, but there was no difference at high concentrations (400 and 800 mg/L; *p* > 0.05). For the recombinant strains, there was no difference in the reduction percentage at an early stage (*p* > 0.05), which might be attributed to the need for recombinant strains to consume part of the protein to tolerate Cr(VI) and adapt to the Cr(VI)-containing environment and then use the excess protein to reduce Cr(VI) to Cr(III). After 12 h of cultivation, recombinant strain 2987 had a slightly higher reduction percentage than 3015, 0415, and 3237 (*p* < 0.05). These results indicated that the reduced ability of the strains to Cr(VI) was M52 ≈ 2987 > 3015 > 0415 ≈ 3237, suggesting that these four functional genes, among which orf2987 had the greatest reducing capability, might play a role in reducing Cr(VI) in M52. However, when combined with the results of the Cr(VI) tolerance analysis for recombinant strains, orf3015, orf0415, and orf3237 were not substantially different from orf2987 in Cr(VI) tolerance, but considerably different in Cr(VI) reduction. As a result, Cr(VI) tolerance of orf2987 might be due to its Cr(VI) reduction abilities, and orf2987 may be a chrome-reduction gene, which were consistent with previous genomic (Li et al., 2021) and proteome (data being submitted) analyses.

### SEM-EDS Analysis

**Figure 5:**
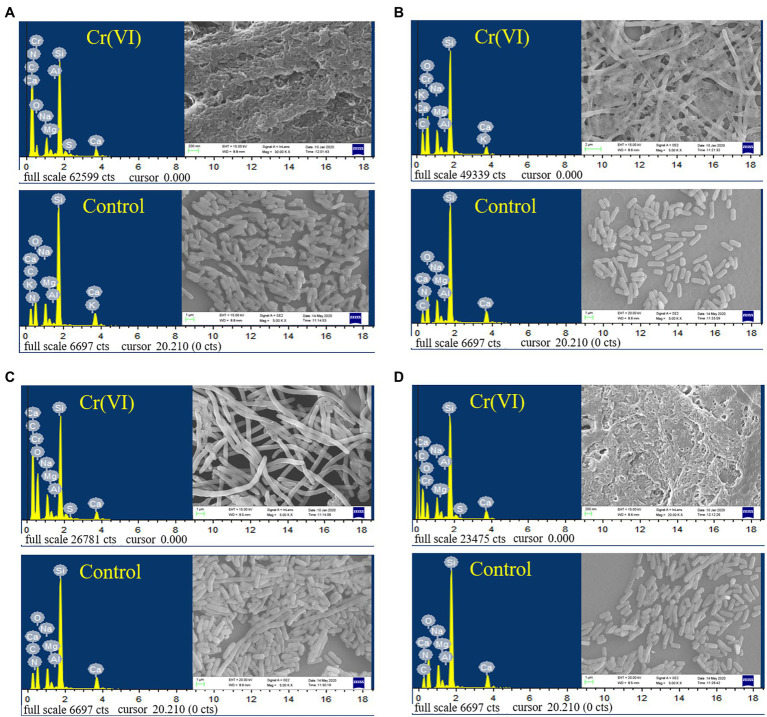
SEM images and EDS analysis of recombinant strains 2987 **(A)**, 3015 **(B)**, 0415 **(C)** and 3237 **(D)**. LB medium containing 100 mg/L Cr(VI) was used as the experimental group, and medium without 100 mg/L Cr(VI) was used as the control.

As shown in Figure 5, the recombinant strains 2987, 3015, 0415 and 3237 were all smooth, short, rod-shaped strains with blunted ends at 5000x magnification. However, the morphology of recombinant strains treated with Cr(VI) was altered in comparison to that of the Cr(VI)-free group (control). After treatment with Cr(VI), the recombinant strain 0415 (Figure 5C) was converted from a short rod shape to a narrow rod shape, but the strain remained smooth, and no adhesion was formed between the strains. However, the morphology of recombinant strains 2987, 3015 and 3237 all changed significantly. There was no improvement in strain volume when recombinant strains 2987 (Figure 5A) and 3237 (Figure 5D) were treated with Cr(VI), but the surface of the strain became rough and the strains adhered to each other. The length of recombinant strain 3015 (Figure 5B) increased to 50 μm after treatment with Cr(VI), and the strains exhibited a form of mutual adhesion. The morphology of the recombinant strains changed under chromium stress, and its surface became rough instead of smooth, which might be due to chromium reduction product adsorption on its surface. In response to the danger of toxic pollutants, Karthik (Karthik et al., 2017) discovered the similar phenomenon in the *Cellulosimicrobium funkei* strain AR8 that bacteria would congregate together, and undertake morphological modifications to defend themselves. The SEM micrograph showed that recombinant strain 0415 was the least affected by Cr(VI), while recombinant strains 2987 and 3237 were the most severely affected. With the exception of 2987, the resistance of recombinant strains to Cr(VI) was consistent with the previous results of 3015 ≈ 0415 > 3237. This showed that while 2987 would tolerate and reduce Cr(VI) well within 72 h, the strain damage was severe and might not have a long-term sustained recovery effect. These results supported our hypothesis that the primary function of orf2987 in M52 was to decrease Cr(VI) rather than to protect the strain. The recombinant strain 0415 exhibited the greatest capacity to resist Cr(VI) in terms of strain shape and structure. Therefore, combined with the results of Cr(VI) tolerance and reduction experiment, orf0415 may primarily play the protective function of Cr(VI) tolerance in M52, rather than the reduction role, which may be mostly performed by orf2987.

The EDS spectra of the elementary analysis of the recombinant strains before and after the reduction of Cr(VI) are shown in Figure 5. Cr(VI) was not detected before the treatment (control), and characteristic peaks of Cr were observed after reduction in all recombinant strains, which was consistent with the findings of Vidyalaxmi et al. (2019). Some coexisting chemical elements have been observed, including C, O, Na, Mg, Al, Si, S, and Ca, similar to Zeng et al. (2019). EDS element mapping (Supplementary Figure S3) revealed that the surface of all recombinant strains consisted mostly of C and O elements that were scattered throughout the growth of the strain and contained a trace of Cr. As a result, it was suggested that, the recombinant strains had a weak capacity to adsorb Cr. This might be because the surface of *E. coli* contains hydroxyl, carbonyl, carboxyl, sulfonate, amide, imidazole, phosphonate, and phosphate diester functional groups, which can interact with metal ions (Quintelas et al., 2009). However, whether the Cr adsorbed was Cr(VI) or Cr(III) must be determined using XRD, FT-IR, and XPS.

### XRD, FT-IR, and XPS Analysis

**Figure 6:**
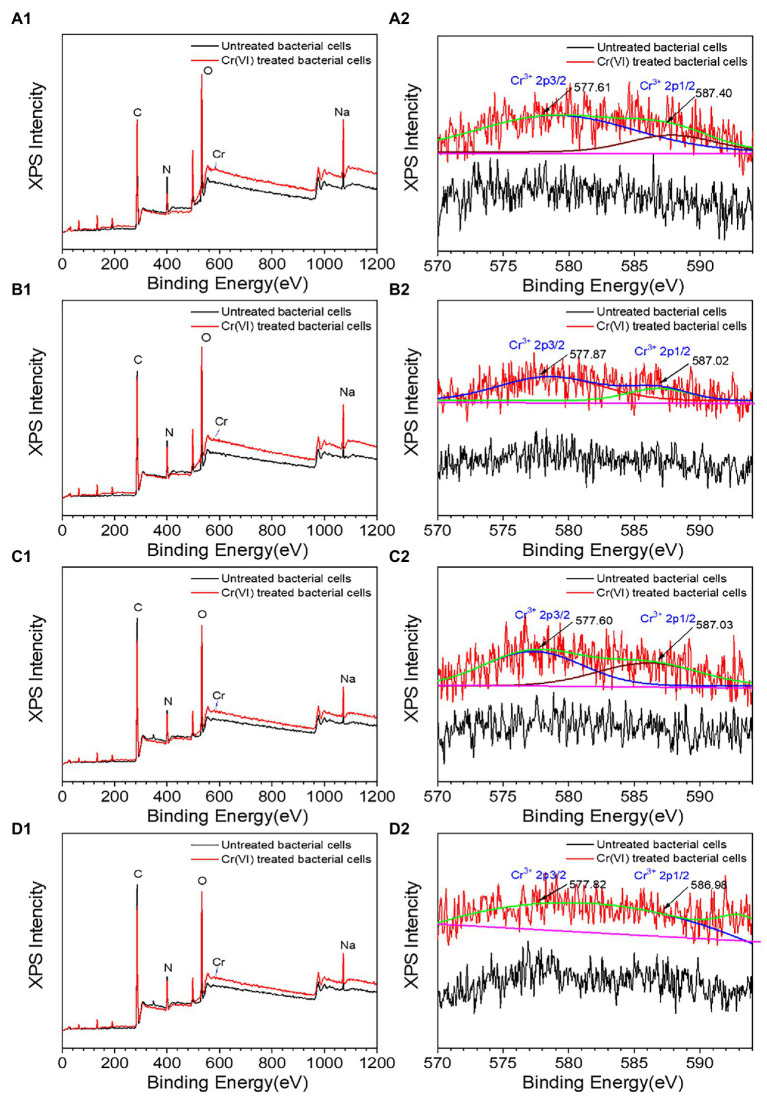
XPS spectra of recombinant strains 2987 **(A1,A2)**, 3015 **(B1,B2)**, 0415 **(C1,C2),** and 3237 **(D1,D2)**. LB medium containing 100 mg/L Cr(VI) was used as the experimental group, and medium without 100 mg/L Cr(VI) was used as the control.

Cr was observed on the surface of all recombinant strains following treatment with Cr(VI), as shown in Figures 6A1–D1, but the specific changes were different. The XRD diffraction peak (Supplementary Figure S4a) with recombinant strain 2987 was concave at 28.11°, with the diffraction peak occurring at 21.82°, suggesting that the surface structure of the strain had changed. The FT-IR results (Supplementary Figure S4b), an important method for defining functional groups that interact with metals and strains (Karthik et al., 2017), showed that the peaks of recombinant strain 2987 at 992, 1087, 1237 and 1358 cm^−1^ vanished after Cr(VI) treatment, while peaks at 2859 and 2930 cm^−1^ shifted, indicating that the strain would reduce Cr(VI) by surface phosphate groups, peptide bonds, carboxyl groups, C-N bonds, and N-H bonds, while alkyl and hydroxyl groups combined with Cr(VI). Further XPS analysis, including the C peak analysis of the strain without Cr(VI) and Cr(VI) culture, is shown in Supplementary Figure S5a. The characteristic peaks of C 1 s were 284.74 eV (C-C or C-H), 285.81 eV (C=N) and 287.90 eV (C=O). The peaks of O 1 s (Supplementary Figure S5b) were 531.14 eV (O=C) and 532.24 eV (C-OH). The characteristic peak results of C 1 s and O1s are consistent with those of Li et al. (2016). The weak peaks of Cr 2p and Cr 3p (Figure 6A2) were situated at 577.61 and 587.40 eV, which were compatible with the Cr(III) characteristic peaks (Cheng et al., 2016). The results indicated that the recombinant strain 2987 reduced Cr(VI) to Cr(III) by providing electrons from amide, carboxyl and hydroxyl functional groups, and reduction product existed on the cell surface. But XRD diffraction peaks are inconsistent with characteristic peaks such as Cr_2_O_3_, Cr(OH)_3_ and other inorganic crystalline compound (Karthik et al., 2017; Tan et al., 2020), suggesting that the reduced products may reside on the surface of the strain in the form of organic complexes that require further identification (Li et al., 2019). Our previous research discovered that M52 may engage in Cr(III) binding *via* amide, carboxyl, hydroxyl, alkyl and phosphorylated functional groups on the cell surface (Li et al., 2021). Similar to our results, Huang et al. (2021) also found that hydroxyl, carboxyl and phosphorylated functional groups were involved in Cr(III) binding in *Sporosarcina Saromensis* W5. The recombinant strain 2987 possessed amide, carboxyl and hydroxyl, but lacked alkyl and phosphate functional groups. The above results indicated that orf2987 was important in reducing Cr(VI) by M52, but the reduction process was not solely dominated by orf2987.

The XRD results of recombinant strains 3015 (Supplementary Figure S4c), 0415 (Supplementary Figure S4e) and 3237 (Supplementary Figure S4g) were altered after treatment with Cr(VI). The diffraction peaks of recombinant strain 3015 and 0415 disappeared at 10.74° and 21.82°, while a new diffraction peak of 17.50° appeared in recombinant strain 3015. But recombinant strain 3237 was different, since the diffraction peaks at 10.74° and 25.14° vanished. These results suggested that the surface structure of the three recombinant strains had changed. But these peaks are not consistent with Cr(III) crystalline compound characteristic peaks. The FT-IR spectrum showed that the peaks of recombinant strain 3015 (Supplementary Figure S4d) at 997, 1,088, 1,239, 1,396, and 1,451 cm^−1^ vanished after treatment with Cr(VI), recombinant strain 0415 (Supplementary Figure S4f) showed that the peaks at 987 and 1,082 cm^−1^ disappeared, and recombinant strain 3237 lack of peaks at 1,359 and 1,456 cm^−1^ in (Supplementary Figure S4h), as well as a shift in the relative intensity of the peak at 2,931 cm^−1^. In order to better identify functional groups, XPS detection was used to showed that the characteristic peaks of recombinant strain 3015 (Supplementary Figures S5c,d) and 0415 (Supplementary Figures S5e,f) at C 1 s and O 1 s were the same as those of 2987, and the characteristic peaks of Cr 2p and Cr 3p showed that recombinant strains 3015 (Figure 6B2) and 0415 (Figure 6C2) could fit the characteristic peaks of Cr(III). The characteristic peaks of recombinant strain 3237 at C 1 s and O 1 s matched those of the other three recombinant strains, as shown in Supplementary Figures S4g,h, but there was no characteristic Cr peak when fitting Cr 2p and Cr 3p (Figure 6D2). These results indicated that phosphate, alkyl, amide, carboxyl and hydroxyl were involved in the reduction of Cr(VI) and combination of Cr(III) in recombinant strain 3015 and 0415, but nitrate, amide, carboxyl and hydroxyl and alkyl only were involved in the reduction of Cr(VI) in recombinant strain 3237. The functional groups involved in the reduction of Cr(VI) and combination of Cr(III) in recombinant strains 3015 and 0415 were similar to M52 (Li et al., 2021), and these groups have been proven to have the same function in other strains (Chen et al., 2019; Kumar and Dwivedi, 2019; Huang et al., 2021). Alkyl and phosphate functional groups were lacked in the recombinant strain 2987 but were present in 3015 and 0415, which prompted that orf3015 and orf0415 might play a role in the binding reduction product of M52 alongside orf2987.

When the results of chromium tolerance, chromium reduction, and characterization analysis were combined, it was evident that the four functional genes did not adsorb Cr(VI), but rather reduced Cr(VI) before adsorbing its reduction product, with orf2987 mostly reducing Cr(VI) in M52 and orf0415 primarily protecting the strain. After reducing Cr(VI), the orf2987 and orf0415 may interact with the reduced products *via* amide, carboxyl, hydroxyl, alkyl, and phosphorylation functional groups. However, orf3015 and orf3237 played a weaker role in these processes. This implies that orf2987 and orf0415 were the main functional genes in M52. Therefore, in the subsequent experiments, we concentrated on the two recombinant strains 2987 and 0415 to investigate the best conditions for the expression of the 2987 and 0415 proteins.

### Effect of pH and Temperature on Cr(VI) Reduction

**Figure 7:**
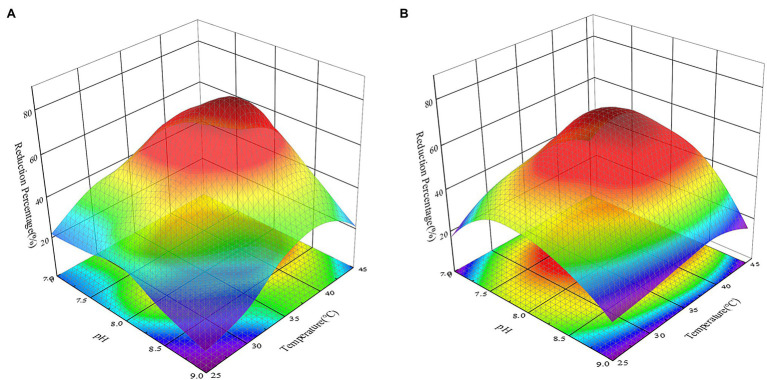
Response surface 3D plot of pH versus temperature showing the effect of independent variables on the reduction of Cr(VI) by recombinant strains 2987 **(A)** and 0415 **(B)** at 48 h. The higher the reduction rate is, the more dark red the color appears; the lower the reduction rate is, the more dark blue the color appears. Data represent means ± SEM. All experiments were repeated three times.

The activity of reductase, the availability of heavy metal ions, and the active sites of heavy metal binding on the cell surface could all be affected by pH and temperature, reducing the effectiveness with which heavy metals are removed by microorganisms (Wu et al., 2019). At the same time, Khattar et al. (2014) proved that acidic conditions could impact the structure of microbial sulfate transporters, inhibiting the removal of Cr(VI) by strains. Many studies have investigated the effects of temperature or pH on the removal of heavy metals by bacteria as two separate parameters (Khattar et al., 2014; Wu et al., 2019). In this study, however, the factorial study revealed that pH and temperature influenced the reduction of Cr(VI) by all recombinant strains and that there was an association between the two (*p* < 0.05). Gruber et al. (2016) also discovered that considering temperature and pH independently will exaggerate their influence effect when compared to considering their interaction. This might be because when the temperature of an alkaline solution rises, the K_w_ of the solution rises, causing the concentration of c(H^+^) in the solution to rise and the pH of the solution to decrease. The effect of pH and temperature on the reduction of Cr(VI) by strains must therefore be thoroughly explored. As shown in Figure 7, changing the pH and temperature had an influence on the recombinant strains 2987 and 0415. Previous study had showed that M52 could resist pH 7.0–8.5 and temperatures 25–35°C, with pH 8.0 and temperatures 35°C being the optimal pH and temperature (Zhao et al., 2016). Recombinant strains 2987 and 0415 were able to survive at pH 7.0–9.0 and temperatures 25–45°C, with 7.5–8.5 and 37°C being the optimum pH and temperature, respectively, and the former showed higher environmental tolerance than M52.

From a different angle, the reduction percentage of Cr(VI) by recombinant strains was lower at both ends of the pH and temperature gradient (Figure 7). From the perspective of pH, this may be due to an inappropriate acid-based environment that harmed growth, metabolic activity, and Cr(VI)-reducing recombinant strains (Xu et al., 2012). High temperatures could induce changes in ribosomal conformation and loss of Cr(VI)-reductase activity, as well as changes in membrane structure or protein synthesis inactivation, while low temperatures considerably decrease cell fluidity, preventing the transport system from functioning (Narayani and Shetty, 2013; Das et al., 2014; Karthik et al., 2017).

### Effect of Metal Ions and Small Molecules on Cr(VI) Reduction

**Figure 8:**
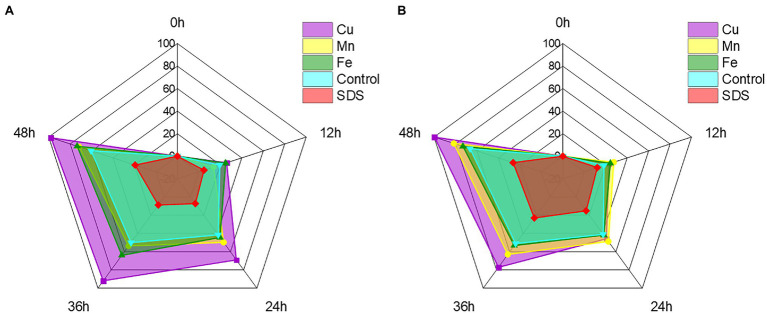
Radar chart of metal ions and small molecules showing the effect on the reduction of Cr(VI) by recombinant strains 2987 **(A)** and 0415 **(B)**. The shaded area is used to indicate the strain’s ability to reduce Cr(VI). Data represent means ± SEM. All experiments were repeated three times.

With the exception of Cr(VI), certain other heavy metal ions and small molecules may also affect the ability of recombinant strains to reduce Cr(VI) in industrial wastewater (Wani et al., 2019; Tan et al., 2020). Cu^2+^, Mn^2+^, and Fe^2+^ are frequently present with Cr(VI) in wastewater from the electroplating and mining industries, whereas SDS, as detergent and textile auxiliaries, is present with Cr(VI) in textile wastewater (Shadrunova and Orekhova, 2015; Khosravi et al., 2020). These heavy metal ions and small molecules may impair the recombinant strain’s capacity to remove Cr(VI) in practice, thus it is critical to test the impact of Cr(VI) removal prior to application. To further improve the reaction conditions, the effects of typical heavy metal ions such as Cu^2+^, Mn^2+^ and Fe^2+^, as well as small molecules, were investigated on the reduction effect of Cr(VI). The recombinant strain 2987 and 0415 rapidly reduced Cr(VI) by 100 mg/L over time, with the rates reaching a limit of 48 h. It was clear from the control that Cu^2+^, Fe^2+^, and Mn^2+^ could all support the reduction of Cr(VI) to differing degrees, with Cu^2+^ having the greatest promotional effect (*p* < 0.05; Figure 8). Recombinant strain 2987 and 0415 completely reduced 100 mg/L Cr(VI) by adding Cu^2+^. Tanrikulu et al. (2011) showed that Cu^2+^ was an essential component of antioxidant enzymes such as catalase and superoxide dismutase as well as the prosthetic group of many reductases (Abe et al., 2001). In addition, Xu et al. (2015) also observed that Cu^2+^ was an essential component of electron transfer in the oxidative respiratory system. Therefore, it was inferred that Cu^2+^ could improve the Cr(VI)-reduction capacity by enhancing the resistance of the recombinant strains to Cr(VI) and the electron transfer efficiency. Fe^2+^ and Mn^2+^ could also promote Cr(VI) reduction by recombinant strain 2987 and 0415, but the strength of the promotion varied by strain. The promotional impact of metal ions on recombinant strain 0415 was Cu^2+^ > Mn^2+^ > Fe^2+^ (*p <* 0.05), whereas Cu^2+^ > Fe^2+^ > Mn^2+^ was the impact on recombinant strains 2987 (*p* < 0.05). Bansal et al. (2019) assumed that Fe^2+^, as an electron donor, may play an important role in Fe/Cr redox coupling. Mn^2+^ was nearly related to the cycle of every other element and had an intimate relationship with the wellbeing, metabolism, and function of microorganisms (Hansel, 2017). Thus, Fe^2+^ and Mn^2+^ can serve as Cr(VI)-reductase activators to promote the reduction of Cr(VI) by recombinant strains. In comparison to metal ions, SDS inhibited the reduction of Cr(VI) by recombinant strains, which was the same as the study of Wani et al. (2019). This may be because SDS could damage the cell wall and denature the protein (Krainer et al., 2020), affecting the resistance of the strain to Cr(VI) and the activity of Cr(VI)-reductase. In general, Cu^2+^, Fe^2+^ and Mn^2+^ could increase the Cr(VI) reduction efficiency of the recombinant strains 2987 and 0415, with Cu^2+^ being the most effective, whereas SDS could decrease the Cr(VI) reduction efficiency of the recombinant strains 2987 and 0415.

## Conclusion

This study confirmed that functional gene segments orf2987, orf3015, orf0415, and orf3237 all had the ability to tolerate and reduce Cr(VI), with orf2987 exhibiting primarily reduction characteristics, orf0415 exhibiting primarily tolerance characteristics, while orf3015 and orf3237 exhibiting only minor effects on both aspects. Recombinant strains 2987 and 0415 could reduction Cr(VI) *via* amide, carboxyl, hydroxyl, alkyl and phosphorylation functional, and both could combine with the reduced product, Cr(III) organic complexes, on the cell surface, with 2987 being more competent. The optimal pH of recombinant strains 2987 and 0415 was 7.5–8.5, while the optimal temperature was 37°C, demonstrating higher environmental tolerance than M52. Cu^2+^, Fe^2+^, and Mn^2+^ promote the strains, with Cu^2+^ being the most, whilst SDS may inhibit them. Although this research provided a good theoretical foundation for investigating the functional genes and reduction mechanism of M52 to reduce Cr(VI), it is likely that a single gene fragment will be unable to replace the combined effect of numerous gene clusters. It also indicates that further research may be conducted to create recombinant strains with combined effectiveness to Cr(VI) reduction efficiency.

## Data Availability Statement

The raw data supporting the conclusions of this article will be made available by the authors, without undue reservation.

## Author Contributions

QA: writing—original draft, formal analysis, validation, and visualization. MZ: conceptualization, investigation, validation, and visualization. DG and CF: methodology and data curation. GW and HX: validation and visualization. JL: validation and investigation. WZ and YL: investigation. XC: methodology. WY: validation. RZ: conceptualization, methodology, resources, supervision, project administration, and writing—review and editing. All authors contributed to the article and approved the submitted version.

## Funding

This work was supported by the National Natural Science Foundation of China (NSFC No. 81673129), the Education Scientific Research Project of Young Teachers in Fujian Province (No. JAT160001), and the College Student Innovation and Entrepreneurship Training Program Support Project of Xiamen University (No. 2019X0528).

## Conflict of Interest

The authors declare that the research was conducted in the absence of any commercial or financial relationships that could be construed as a potential conflict of interest.

## Publisher’s Note

All claims expressed in this article are solely those of the authors and do not necessarily represent those of their affiliated organizations, or those of the publisher, the editors and the reviewers. Any product that may be evaluated in this article, or claim that may be made by its manufacturer, is not guaranteed or endorsed by the publisher.
