# Supplementary material for: Cr(VI) Removal by Recombinant Escherichia coli Harboring the Main Functional Genes of Sporosarcina saromensis M52
Source: Front Microbiol. 2022 Mar 3;13:820657. doi: 10.3389/fmicb.2022.820657 (PMC8927625; doi:10.3389/fmicb.2022.820657)
Supplement: Supplementary file 1 [file Table_1.DOCX]

**Cr(VI) removal by recombinant *E. coli* harboring the main functional genes of *Sporosarcina saromensis* M52**

**Qiuying An^1,†^, Min Zhang^2,†^, Dongbei Guo^1^, Guangshun Wang^1^, Hao Xu^1^, Chun Fan^1^, Jiayao Li^3^, Wei Zhang^1^, Yi Li^1^, Xiaoxuan Chen^1^, Wanting You^1^, Ran Zhao^1,*^**

^1^ State Key Laboratory of Molecular Vaccinology and Molecular Diagnostics, School of Public Health, Xiamen University, Xiamen 361102, Fujian, China

^2^ Huzhou Center for Disease Prevention and Control, Huzhou 313000, Zhejiang, China

^3^ National Cancer Center/National Clinical Research Center for Cancer/Cancer Hospital & Shenzhen Hospital, Chinese Academy of Medical Sciences and Peking Union Medical College, Shenzhen 518116, Guangdong, China

**Table S1** Primer pairs sequences

| Primer name | Primer sequences (5′→3′) | Restriction enzyme site |
| --- | --- | --- |
| Ex2987-F | CATATG**GGATCC**ATGACAATTACAGTTAAAGCAATT | *EcoR* Ⅰ |
| Ex2987-R | CCC**AAGCTT**TTATGCAGAAACAGTTGTTTT | *Hind* Ⅲ |
| Ex3015-F | GACAC**CCATGG**GGATGACAGATAAATACGCAATACT | *Nco* Ⅰ |
| Ex3015-R | GTGTC**CTCGAG**TCACACAA | *Xho* Ⅰ |
| Ex0415-F | GACAC**CCATGG**GGATGAACGAGCAGGCACTGTCTATCC | *Nco* Ⅰ |
| Ex0415-R | GTGTC**CTCGAG**TTACTGTTCTACTTCAACGGACGTTTCT | *Xho* Ⅰ |
| Ex3237-F | CCC**AAGCTT**ATGGTCATCGAATTATTAACTGCA | *Hind* Ⅲ |
| Ex3237-R | GTGTC**CTCGAG**TCAGTTCATTTCAAAACCTTGT | *Xho* Ⅰ |

Note: The bold portion is the restriction site. The underlined portion is the protected base.
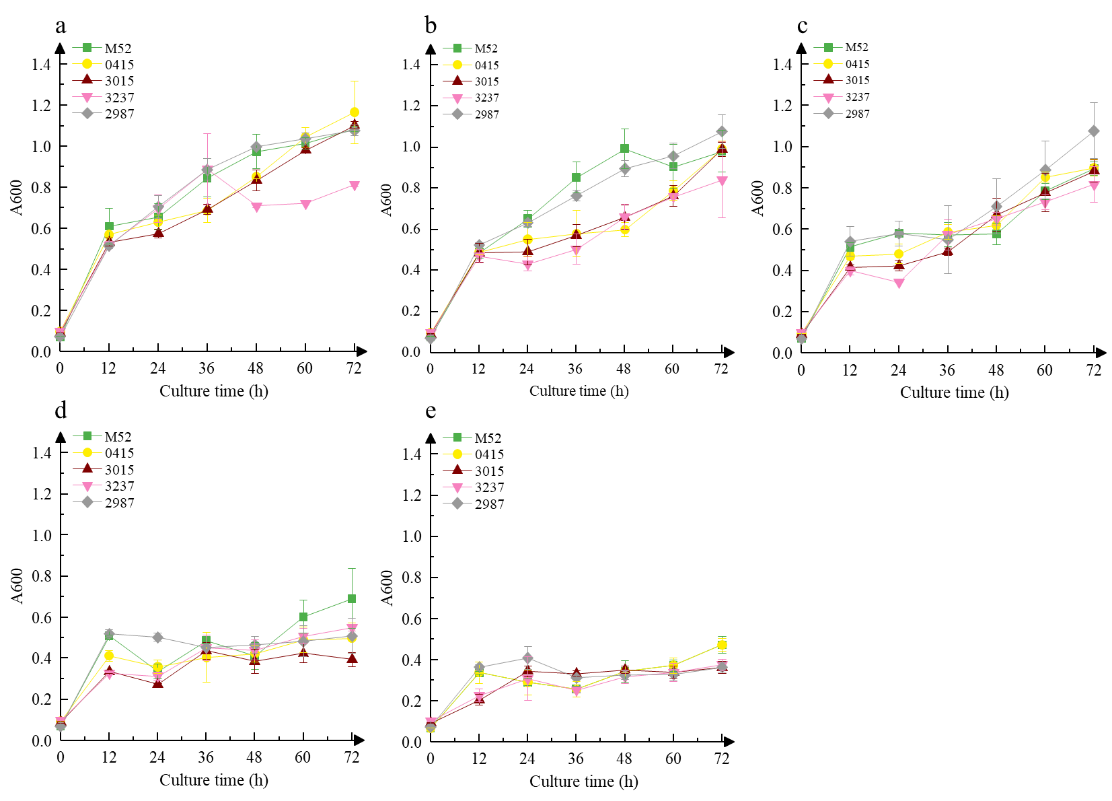


**Fig. S1.** The growth of all strains in different concentrations of Cr(VI).

a: 50 mg/L Cr(VI) b: 100 mg/L Cr(VI) c: 200 mg/L Cr(VI) d: 400 mg/L Cr(VI) e: 800 mg/L Cr(VI).


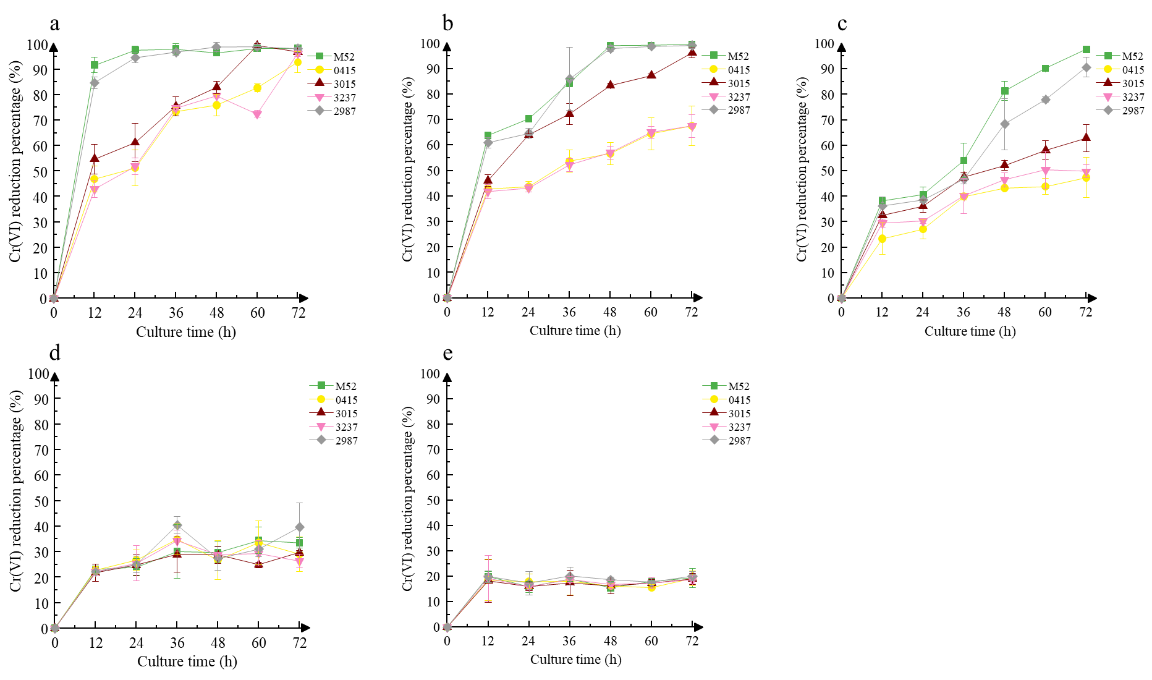


**Fig. S2.** The reduction percentage of all strains under different concentrations of Cr(VI).

a: 50 mg/L Cr(VI) b: 100 mg/L Cr(VI) c: 200 mg/L Cr(VI) d: 400 mg/L Cr(VI) e: 800 mg/L Cr(VI).


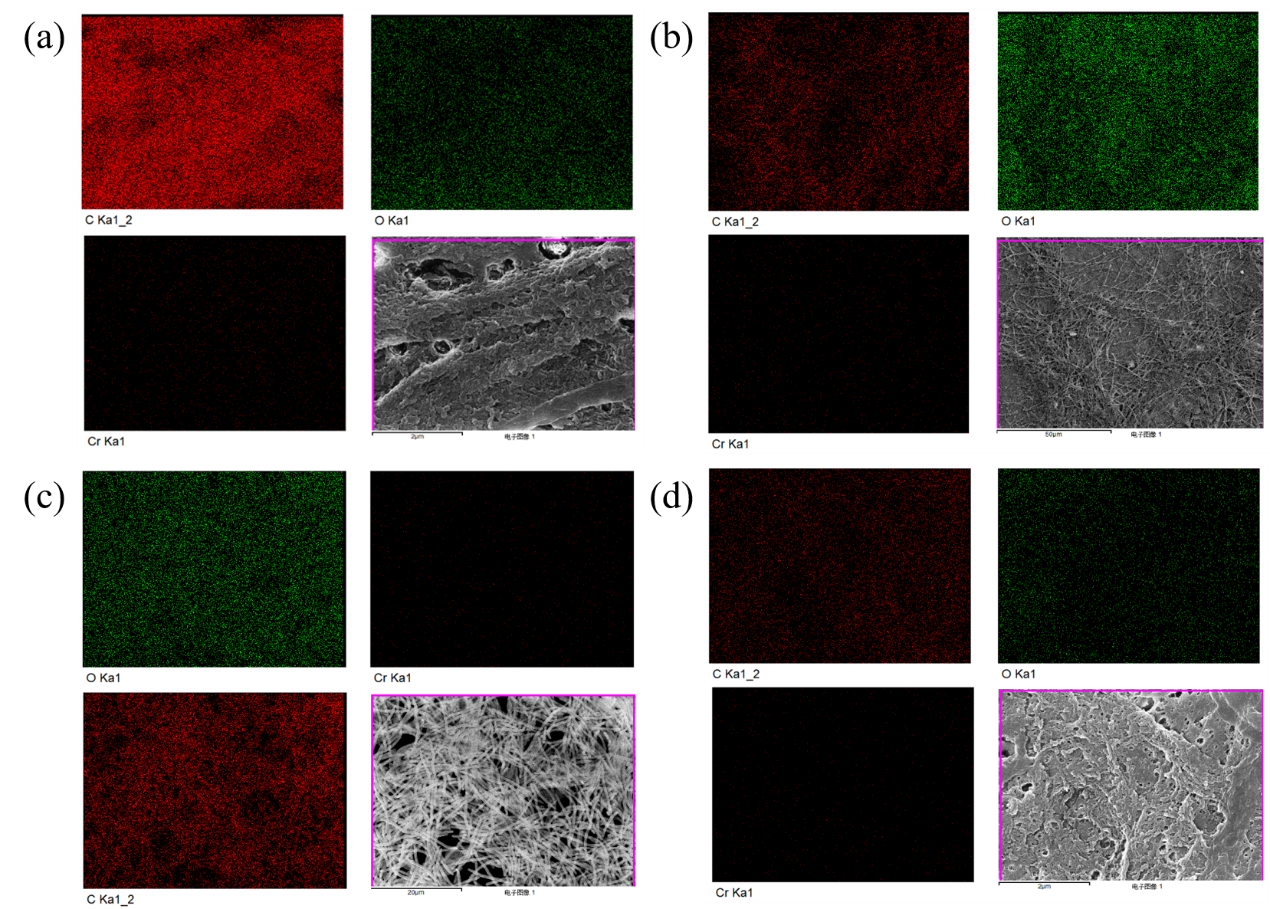


**Fig. S3.** SEM-EDS element map of recombinant strain 2987(a), 3015(b), 0415(c) and 3237(d).


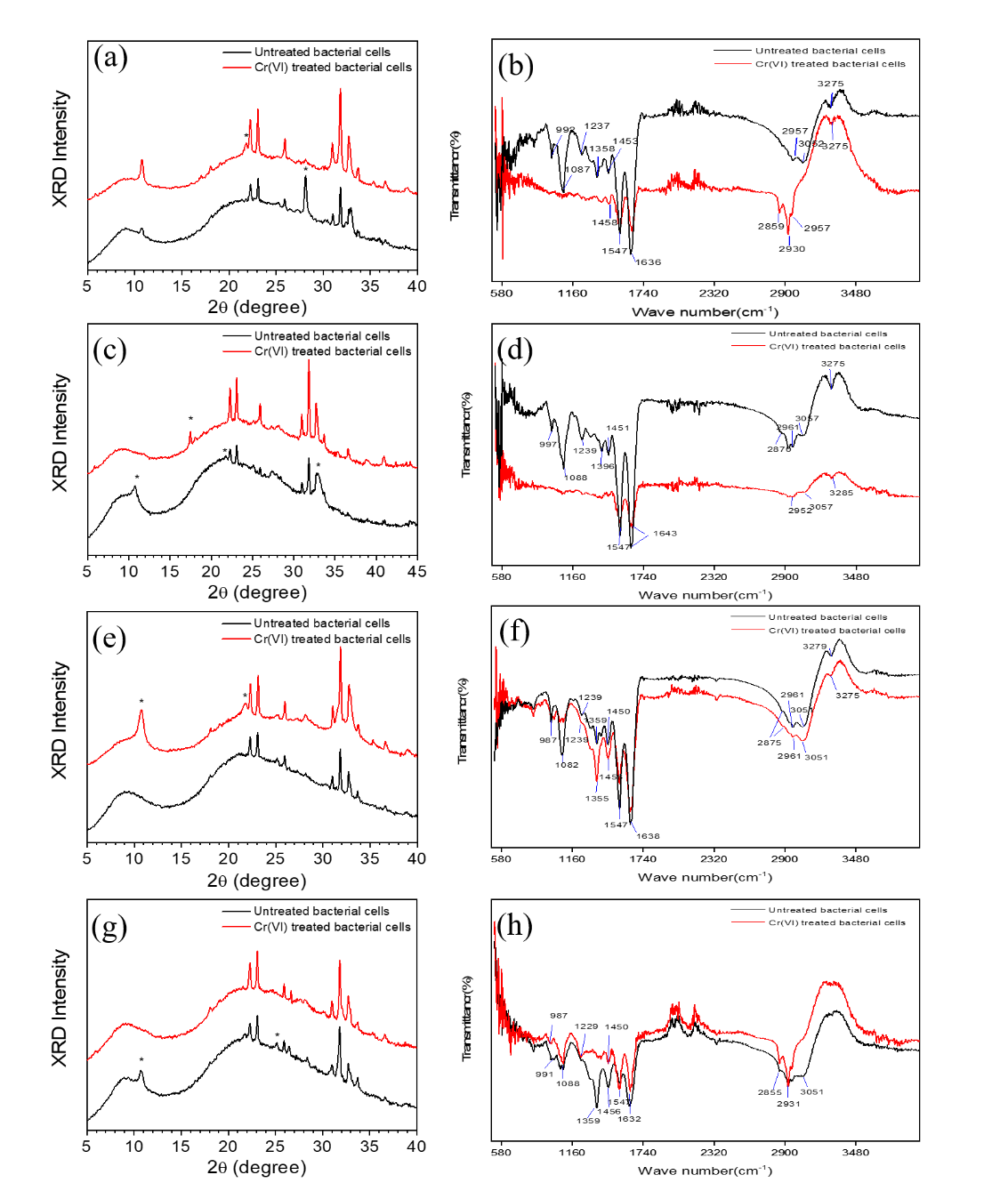


**Fig. S4.** The XRD biomass spectrum and the FT-IR spectrum of recombinant strains 2987(a b), 3015(c d), 0415(e f) and 3237(g h) obtained in LB medium with or without Cr(VI) at an initial Cr(VI) concentration of 100 mg/L.


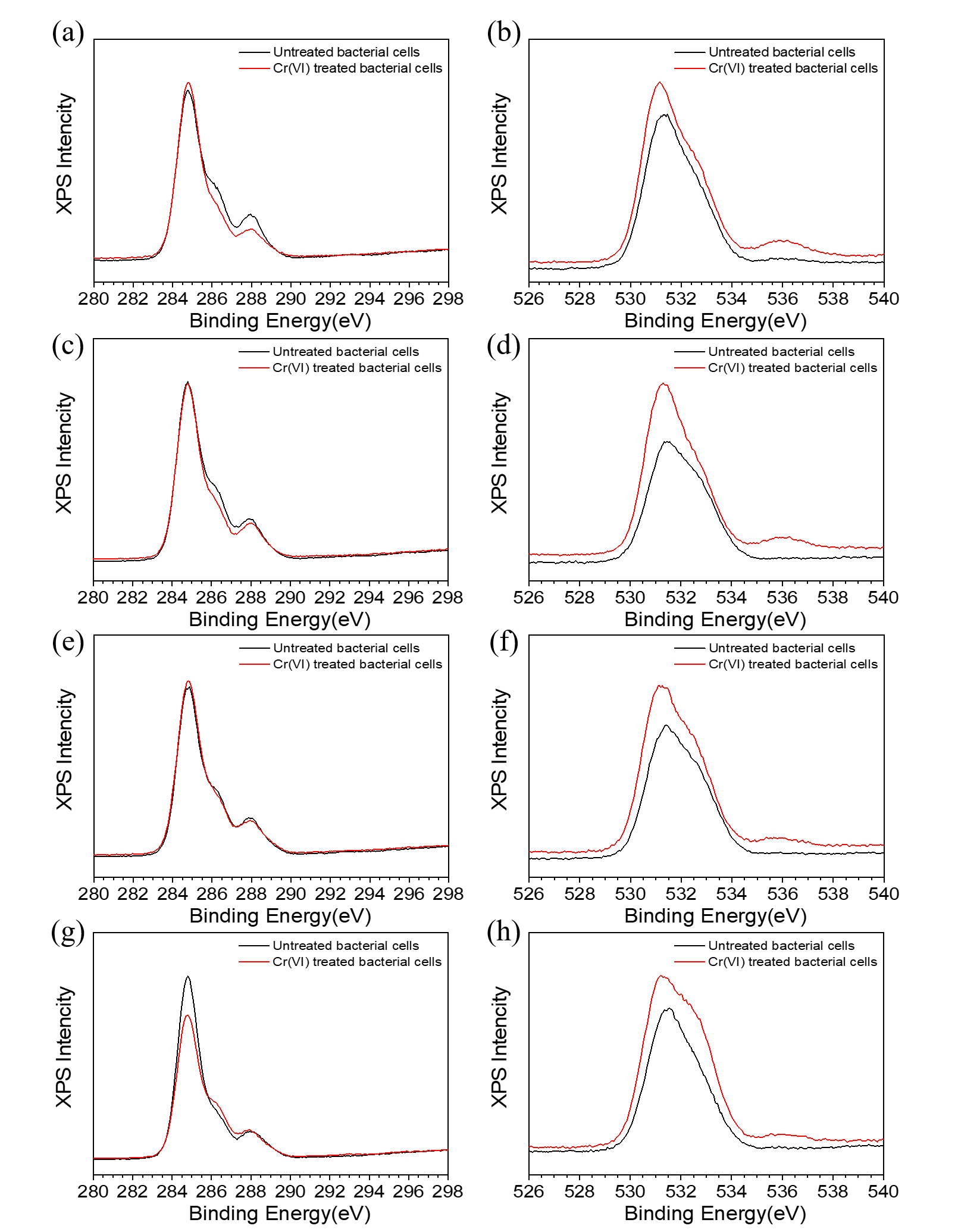


**Fig. S5.** The characteristic peaks of recombinant strain 2987, 3015, 0415 and 3237 at C 1s and O 1s. (a): C 1s characteristic peak of recombinant strain 2987 (b): O 1s characteristic peak of recombinant strain 2987 (c): C 1s characteristic peak of recombinant strain 3015 (d): O 1s characteristic peak of recombinant strain 3015 (e): C 1s characteristic peak of recombinant strain 0415 (f): O 1s characteristic peak of recombinant strain 0415 (g): C 1s characteristic peak of recombinant strain 3237 (h): O 1s characteristic peak of recombinant strain 3237.
